# Urothelial bladder cancer may suppress perforin expression in CD8^+^ T cells by an ICAM-1/TGFβ2 mediated pathway

**DOI:** 10.1371/journal.pone.0200079

**Published:** 2018-07-02

**Authors:** Ciputra Adijaya Hartana, Emma Ahlén Bergman, A. Ali Zirakzadeh, David Krantz, Malin E. Winerdal, Max Winerdal, Markus Johansson, Farhood Alamdari, Tomasz Jakubczyk, Hans Glise, Katrine Riklund, Amir Sherif, Ola Winqvist

**Affiliations:** 1 Department of Medicine Solna, Unit of Immunology and Allergy, Karolinska Institutet, Stockholm, Sweden; 2 Department of Surgical and Perioperative Sciences, Urology and Andrology, Umeå University Hospital, Umeå, Sweden; 3 Department of Urology, Sundsvall Hospital, Sundsvall, Sweden; 4 Department of Urology, Västmanland Hospital, Västerås, Sweden; 5 Department of Urology, Länssjukhuset Ryhov, Jönköping, Sweden; 6 Department of Radiation Sciences, Umeå University, Umeå, Sweden; Mie University Graduate School of Medicine, JAPAN

## Abstract

The immune system plays a significant role in urothelial bladder cancer (UBC) progression, with CD8^+^ T cells being capable to directly kill tumor cells using perforin and granzymes. However, tumors avoid immune recognition by escape mechanisms. In this study, we aim to demonstrate tumor immune escape mechanisms that suppress CD8^+^ T cells cytotoxicity. 42 patients diagnosed with UBC were recruited. CD8^+^ T cells from peripheral blood (PB), sentinel nodes (SN), and tumor were analyzed in steady state and *in vitro*-stimulated conditions by flow cytometry, RT-qPCR, and ELISA. Mass spectrometry (MS) was used for identification of proteins from UBC cell line culture supernatants. Perforin was surprisingly found to be low in CD8^+^ T cells from SN, marked by 1.8-fold decrease of *PRF1* expression, with maintained expression of granzyme B. The majority of perforin-deficient CD8^+^ T cells are effector memory T (T_EM_) cells with exhausted Tc2 cell phenotype, judged by the presence of PD-1 and GATA-3. Consequently, perforin-deficient CD8^+^ T cells from SN are low in T-bet expression. Supernatant from muscle invasive UBC induces perforin deficiency, a mechanism identified by MS where ICAM-1 and TGFβ2 signaling were causatively validated to decrease perforin expression *in vitro*. Thus, we demonstrate a novel tumor escape suppressing perforin expression in CD8^+^ T cells mediated by ICAM-1 and TGFβ2, which can be targeted in combination for cancer immunotherapy.

## Introduction

Urinary bladder cancer is one of the most common cancers (estimated 429,800 new cases worldwide). The majority of the cases occur in men and in the developed countries [1]. The pathological feature of urinary bladder cancer is dominated by urothelial bladder cancer (UBC) (90%) [2]. The mortality of UBC is mainly due to tumor invasion beyond the basement membrane into the muscular part of the bladder, which is seen in 20–30% of the UBC cases. This will result in a higher probability of developing cancer metastasis [3].

The main risk factor for developing urinary bladder cancer is tobacco smoking, as well as occupational exposures to irritants [4]. Evidence suggested that parasitic infestation of the urinary bladder with *Schistosoma hematobium* is a major risk factor of urinary bladder squamous cell carcinoma in the Middle East [5]. All these factors are believed to induce a chronic inflammatory environment within the bladder, resulting in a high infiltration of immune cells. These immune cells are responsible of releasing some pro-tumor cytokines and growth factors, which will in turn promote tumor angiogenesis, proliferation of tumor cells, and tumor cells survival [6].

However, despite having tumor-promoting features, the immune cells also possess tumor-suppressive roles in the pathogenesis of UBC. It was demonstrated that high infiltration of T lymphocytes into the tumor correlates positively with UBC patients’ survival [7]. The importance of the immune system in UBC is further demonstrated since intravesicular instillation of Bacillus Calmette Guérin (BCG) vaccine is used as a standard treatment of high grade non-invasive UBC [8]. BCG treatment has been reported to induce an anti-tumor immune reaction, manifested by the effects on T lymphocytes and innate immune cells with promising results in tumor regression [9].

However, based on the Hallmark of Cancer: The Next Generation, cancer cells may escape immune destruction [10]. Several escape mechanisms in avoiding immune destruction have been demonstrated, such as generation of neo-antigens [11, 12] and low expression of MHC class I by tumor cells [13]. Moreover, tumor may create further chronic inflammation that causes prolonged T cell receptors (TCR) engagement (signal 1) and co-stimulatory/co-inhibitory signals (signal 2), with the presence of suppressive cytokines that will induce CD8^+^ T cells exhaustion [14]. Additionally, shift in cytokine dynamics which results in reduced IFNγ and increased IL-4 within this environment will polarize CD8^+^ T cells into low cytotoxic Tc2 cells [15].

In this paper, we focus on the effect of the tumor immune escape on CD8^+^ T cells cytotoxicity in UBC. It is generally known that CD8^+^ T cells have an important role in the defense against tumor cells [16]. The cytosol of CD8^+^ T cells contains granzymes and perforin, stored inside the cytotoxic granules [17]. Upon recognition of tumor cells by CD8^+^ T cells through MHC- tumor peptide complexes, cytotoxic granules will move towards the cell surface and exocytose granzymes and perforin to the immunological synapses [18]. Perforin will in turn form pores in the plasma membrane of tumor cells, allowing entry of granzymes into the cells which then activate the caspases activity, initiating tumor cell apoptosis [19].

To study the phenotype of CD8^+^ T cells from sentinel lymph nodes (SN) is important since it is the first site of interaction between the tumor and the immune system. In most solid cancers, SN will be the first site to receive metastasis from the primary tumors [20]. In this study, we analyzed the impact of tumor-induced immune escape on cytotoxicity and exhaustion of CD8^+^ T cells from peripheral blood (PB), SN, and tumor of the UBC patients.

## Materials and methods

### Patient characteristics and tissue collection

42 patients diagnosed with urothelial bladder cancer staged cTa-cT4aN0M0, were prospectively recruited from four participating hospitals in Sweden (Umeå University Hospital, Sundsvall Hospital, Västerås Central Hospital, and Jönköping/Ryhov Hospital) during the period of 2015–2017 (Table 1). The patients underwent transurethral resection of the bladder (TUR-B) and subsequently radical cystectomy, in accordance with the national guidelines. Peripheral blood (PB) and tumor were collected at TUR-B procedures, whereas PB and sentinel lymph nodes (SN) were collected at cystectomy. Peripheral blood was collected in heparin tubes. SN were detected perioperatively by injection of radioactive tracer ^99m^Technetium followed by detection using gamma probe, as previously described [21]. Lymphadenectomies were then carried out following the detection, in which standard templates of nodal dissection, entailing bilateral obturator and iliac regions, were used. Tumor tissues and lymph nodes were sliced into two parts; one part for immunological analysis in the present study and one part for routine histopathological examination. SN from patient no. 18 and 29 were detected to contain cancer cells by histopathological examination (Table 1). The tissues purposed for immunological analysis were immediately put in RPMI 1640 medium (Sigma-Aldrich) on cold condition (4–8°C) and processed within 24 hours.

**Table 1:** Patients’ characteristics.

Patient number	Age	Gender	Clinical staging	Pathological staging			Specimen received	Neoadjuvant chemotherapy cycles
				pT-stage	pN-stage	pM-stage		
1	56	M	cT2N0M0	pT0	pN0	pM0	TUR-B, Cystectomy	3
2	60	M	cT2N0M0	pT0	pN0	pM0	TUR-B, Cystectomy	3
3	70	F	cT2N0M0	pT2	pN0	pM0	TUR-B, Cystectomy	2
4	70	M	cT2N0M0	pT1	pN0	pM0	TUR-B, Cystectomy	3
5	66	M	cT2N0M0	pTa	pN0	pM0	TUR-B, Cystectomy	3
6	69	M	cT2N0M0	pT3	pN0	pM0	TUR-B, Cystectomy	1
7	68	M	cT2N0M0	pT0	pN0	pM0	Cystectomy	3
8	80	F	cT2N0M0	pT3	pN0	pM0	TUR-B, Cystectomy	0
9	59	F	cT2N0M0	NA^1^	NA^1^	NA^1^	Only TUR-B, no cystectomy	3
10	77	M	cT2N0M0	pTis	pN0	pM0	Cystectomy	3
11	78	M	cTaN0M0G2	NA^1^	NA^1^	NA^1^	Only TUR-B, no cystectomy	0
12	60	M	cT2N0M0	pT2	pN0	pM0	TUR-B, Cystectomy	3
13	72	M	cT3N0M0	pT3	pN0	pM0	Cystectomy	0
14	74	M	cT2N0M0	pT0	pN0	pM0	TUR-B, Cystectomy	3
15	73	M	cT1N0M0G2	NA^1^	NA^1^	NA^1^	Only TUR-B, no cystectomy	0
16	79	M	cT1N0M0G3	NA^1^	NA^1^	NA^1^	Only TUR-B, no cystectomy	0
17	61	M	cT3N0M0	pT3	pN0	pM0	TUR-B, Cystectomy	2
18	71	F	cT2N0M0	pT3	pN1	pM0	TUR-B, Cystectomy	0
19	70	M	cTaN0M0G1	NA^1^	NA^1^	NA^1^	Only TUR-B, no cystectomy	0
20	54	M	cT1N0M0G3	NA^1^	NA^1^	NA^1^	Only TUR-B, no cystectomy	0
21	69	M	cT1N0M0G3	NA^1^	NA^1^	NA^1^	Only TUR-B, no cystectomy	0
22	71	F	cT1N0M0G2	NA^1^	NA^1^	NA^1^	Only TUR-B, no cystectomy	0
23	76	M	cTaN0M0G3	NA^1^	NA^1^	NA^1^	Only TUR-B, no cystectomy	0
24	79	F	cT2N0M0	pT0	pN0	pM0	Cystectomy	0
25	60	F	cT4aN0M0	pT4a	pN0	pM0	Cystectomy	2
26	70	M	cT1N0M0G3	NA^1^	NA^1^	NA^1^	Only TUR-B, no cystectomy	0
27	75	M	cT2N0M0	pT0	pN0	pM0	Cystectomy	3
28	71	M	cT2N0M0G3	pT2	pN0	pM0	TUR-B, Cystectomy	1
29	82	F	cT2N0M0	pT3	pN1	pM0	Only TUR-B, no cystectomy	1
30	70	F	cTaN0M0G2	NA^1^	NA^1^	NA^1^	Only TUR-B, no cystectomy	0
31	73	F	cT3N0M0G3	pT0	pN0	pM0	Only TUR-B, no cystectomy	3
32	77	M	cT1N0M0	NA^1^	NA^1^	NA^1^	Only TUR-B, no cystectomy	0
33	72	M	cT2N0M0	pT2b	pN0	pM0	Only TUR-B, no cystectomy	4
34	61	M	cT1N0M0 + CIS	NA^1^	NA^1^	NA^1^	Only TUR-B, no cystectomy	0
35	79	F	cT2N0M0	pT3b	pN0	pM0	Only TUR-B, no cystectomy	0
36	74	M	cTaN0M0G2	NA^1^	NA^1^	NA^1^	Only TUR-B, no cystectomy	0
37	67	F	cT2N0M0	pT3	pN0	pM0	Only TUR-B, no cystectomy	3
38	79	M	cT1N0M0	NA^1^	NA^1^	NA^1^	Only TUR-B, no cystectomy	0
39	75	M	cTaN0M0G2	NA^1^	NA^1^	NA^1^	Only TUR-B, no cystectomy	0
40	71	F	cT2N0M0	OP^2^	OP^2^	OP^2^	Only TUR-B, no cystectomy	OP^2^
41	84	M	cT2N0M0	OP^2^	OP^2^	OP^2^	Only TUR-B, no cystectomy	OP^2^
42	75	M	cT2N0M0	OP^2^	OP^2^	OP^2^	Only TUR-B, no cystectomy	OP^2^

We recruited 42 patients diagnosed with urothelial bladder cancer. 18 patients underwent TUR-B and radical cystectomy, with cisplatin-based neoadjuvant chemotherapy in between the surgeries. 20 patients did not receive neoadjuvant chemotherapy. Clinical staging was determined post TUR-B and pathological staging was determined post cystectomy.

^1^NA: not applicable. Patients did not receive cystectomy, so pathological staging could not be done.

^2^OP: on progress. Patients have not received neoadjuvant chemotherapy or cystectomy at the end of the study.

Buffy coat specimens from healthy donors, used as controls, were received from the Karolinska University Hospital blood bank. Written consent was obtained from UBC patients in accordance with the Declaration of Helsinki. The study was approved by the regional ethical committee (Etikprövningsnämnden (EPN)–Stockholm, registration number: 2007/71-31, with latest amendment 2017/190-32).

### Single cells isolation

Peripheral blood mononuclear cells (PBMC) were isolated from PB of patients and healthy donors by density gradient separation using Ficoll-Paque Plus (GE Healthcare). PBMC were then suspended in AIM-V medium (Life Technologies).

To isolate tumor infiltrating lymphocytes (TIL), tumor tissue was cut into small pieces and put into C-tubes (Miltenyi) containing 9.8 ml of AIM-V medium, 100 μl DNAse I (10 mg/ml; Sigma Aldrich), and 100 μl of collagenase/hyaluronidase (3000 IU/ml collagenase and 1000 IU/ml hyaluronidase; Stem Cell). The C-tubes were then processed with the GentleMACS (Miltenyi), according to the manufacturer’s instruction. The resulting suspensions were then filtered through 40 μm cell strainer, washed and then suspended in AIM-V medium. To obtain tissue homogenates for lymphocyte activation assay, small part of tumor tissues were sliced and homogenized in 2X phosphate-buffered saline (PBS) by using Bio-Gen PRO200 Homogenizer (Pro Scientific). The tissue homogenates were then heated to 100°C for five minutes to establish protein denaturation.

Single cell suspensions from SN were obtained by gentle pressure homogenization in AIM-V medium through a 40 μm cell strainer. The single cell suspension were then washed and suspended in AIM-V medium.

### Cell culture

To study the functional capacity of the CD8^+^ T cells, isolated lymphocytes from different tissues were cultured for reactivation with tumor homogenates as the antigen source. The cells were cultured *in vitro* in U-bottom 96-well plates (Falcon) with 5x10^5^ total cells per well with 200 μl of total volume in AIM-V medium with the presence of tumor homogenates stimulation in a dilution of 1/100 (v/v). Cells were incubated in a humidified atmosphere with 5% CO_2_ at 37°C. The functional capacity of the cells was analyzed by flow cytometry after seven days of culture. Secreted granzyme B and perforin were analyzed from the culture supernatant at the end of culture using ELISA.

To study the possibility of restoring the cytotoxicity of CD8^+^ T cells, sorted CD8^+^ T cells from SN were cultured *in vitro* in a Tc1 promoting conditions. Cells were stimulated with 5 μg/ml plate-bound anti-CD3 antibody (clone OKT3; Biolegend) and 1 μg/ml soluble anti-CD28 antibody (clone CD28.2; Biolegend) in the medium containing 5 ng/ml recombinant human IL-12 (Calbiochem) and 5 μg/ml neutralizing anti-IL-4 antibody (clone 34019; R&D Systems) with the presence of 100 IU/ml IL-2 (PeproTech). CD8^+^ T cells were cultured in a humidified atmosphere with 5% CO_2_ at 37°C for seven days. The cells were then harvested and analyzed for their cytotoxicity by FACS and RT-qPCR.

To explore the effects exerted by secreted factors of UBC tumor cells on CD8^+^ T cells, sorted CD8^+^ T cells from PB of healthy donors were cultured *in vitro* in the presence of culture supernatants from RT4 (non-muscle invasive; ATCC) and 5637 (muscle invasive; ATCC) UBC cells lines. Initially, both cell lines were cultured in RPMI medium (Sigma Aldrich), supplemented with 10% fetal calf serum (FCS) (Gibco), 1% L-glutamine (Hyclone), and 1% penicillin/streptomycin (Hyclone) at 37°C and 5% CO_2_. Once the cells reached 90% confluency, the conditioned medium was removed and the cell layers were washed three times with PBS and two times with RPMI-Serum and Phenol Red Free Medium (SFM) (Thermo Fisher). The SFM was added to the cells for incubation period of 24 hours, after which the SFM was collected, centrifuged at 1000g for 10 minutes at 4°C, and frozen in -80°C until used. Isolated human CD8^+^ T cells were either cultured in 75% UBC cell line SFM and 25% basal medium (AIM-V) or basal medium (AIM-V) alone. Cells were incubated at 37°C and 5% CO_2_ for five days and harvested for analysis using flow cytometry and RT-qPCR.

### Flow cytometry analysis and sorting

Single cell suspensions isolated from PB, SN, and tumor were stained for surface and intracellular markers for flow cytometry analysis. Briefly, cells were stained with fixable live/dead dye (Life Technologies) to identify dead cells. For cell surface staining, the following fluorochrome-conjugated anti-human antibodies were used: anti-CD45 (clone HI30; BD Biosciences), anti-CD3 (clone UCHT1; BD Biosciences), anti-CD4 (clone RPA-T4; BD Biosciences), anti-CD8 (clone RPA-T8; BD Biosciences), anti-PD1 (clone eBioJ105; eBioscience), anti-CD56 (clone B159; BD Biosciences), anti-CD45RA (clone HI100; BD Biosciences), and anti-CCR7 (clone 150503; BD Biosciences) antibodies. For staining of intracellular markers, cells were fixed and permeabilized using the FOXP3 transcription factor kit (eBioscience), followed by staining with: anti-Granzyme B (clone GB11; eBioscience), anti-Perforin (clone δG9; BD Biosciences), and anti-T-bet (clone 4B10; BioLegend) antibodies. Isotype controls were used for the following markers: Granzyme B, Perforin, T-bet, and PD-1. RT4 and 5637 cell lines were stained in cell suspension with anti-ICAM-1 (clone HA58; BioLegend) and anti-EpCAM (clone 1B7; eBioscience) antibodies. Flow cytometry data were acquired with an LSR Fortessa instrument (BD Biosciences) and analyzed using FlowJo v.10 software (TreeStar).

For cell sorting by flow cytometry, isolated lymphocytes from PBMC, SN, and tumor were stained with fluorochrome-conjugated antibodies using anti-CD8 (clone RPA-T8; BD Biosciences) and anti-56 (clone B159; BD Biosciences) antibodies. Cells were sorted for CD8^+^ T cells defined as CD8^+^CD56^-^. Cell sorting was done using FACS Aria I instrument (BD Biosciences) and FACS Diva software (BD Biosciences). Post-sort purity was more than 85% for all included samples.

### Reverse transcription-quantitative PCR

Messenger RNA (mRNA) was extracted from sorted CD8^+^ T cells using RNeasy Plus Mini kit (Qiagen) according to the manufacturer’s protocols. Extracted mRNA was reverse-transcribed into cDNA after mixing mRNA template with iScript Reverse Transcription Supermix for RT-qPCR (Bio-Rad). RT-qPCR was done with each reaction containing 15.45 ng of cDNA template, 300 nM transcript-specific forward and reverse primers (Eurofins Genomics), and 2X SYBR Select Master Mix (Life Technologies). The primer sequences were: *GZMB* (fwd: 5’-TGCACTG TCATCTTCACCTCT-3’; rev: 5’-CTGTGAAAAGACCCATCCCC-3’), *PRF1* (fwd: 5’-ATGA AGTGGGTGCCGTAGTT-3’; rev: 5’-CAACTTTGCAGCCCAGAAGA-3’), *TBX21* (fwd: 5’-CACTACAGGATGTTTGTGGACGTG-3’; rev: 5’-CCCCTTGTTGTTTGTGAGCTTTAG-3’), *GATA3* (fwd: 5’-AACTGTCAGACCACCACAACCACAC-3’; rev: 5’-GGATGCCTTCCTTCT TCATAGTCAGG-3’), and *RPII* (fwd: 5’-GCACCACGTCCAATGACAT-3’; rev: 5’-GTGCG GCTGCTTCCATAA-3’). Reactions were done for 40 cycles with an initial activation of uracil-DNA glycosylase (UDG) for two minutes in 50°C, AmpliTag DNA polymerase activation for two minutes in 95°C, denaturation in 95°C for 15 seconds, and annealing and extension for one minute in 60°C. RT-qPCR was done in CFX96 Real-Time System (BioRad) and analyzed in CFX Manager Software (BioRad). The relative expression level of target gene transcripts was calculated in respect of internal standard (*RPII*). Expression levels were normalized against control using ΔΔCt calculation.

### ELISA

Culture supernatants were collected at appropriate culture time points, centrifuged in order to remove cell debris and frozen at -80°C until analysis. Secreted soluble granzyme B and perforin were measured by sandwich ELISA kit (Mabtech). MaxiSorp flat-bottom 96-well plates (Nunc) were coated with 2 μg/ml Granzyme B antibody (clone GB10) or 4 μg/ml Perforin antibody (clone Pf-80/164) overnight at 4–8°C. The plates were then washed with PBS and blocked with PBS containing 0.05% Tween 20 (Sigma Aldrich) and 0.1% Bovine Serum Albumin (Sigma Aldrich) for one hour. Samples and standards were then added and incubated for two hours at room temperature, washed and incubated with biotin-conjugated antibodies against of Granzyme B (1 μg/ml; clone GB11) or Perforin (1 μg/ml; clone Pf-344) for one hour at room temperature. Plates were then washed and incubated with 1 μg/ml Streptavidin-HRP for one hour at room temperature followed by another wash. TMB substrate (Mabtech) were then added and incubated for 15 minutes. The reaction was stopped with 0.01 M H_2_SO_4_ and the optical density in 450 nm wavelength were measured by EnSpire 2300 Multilabel Reader (Perkin Elmer). Sample concentrations were determined based on standard curve after subtraction from blank control.

### Supernatant preparation for proteomic analysis

The collected supernatant from RT4 and 5637 cell lines were concentrated using Amicon Ultra-15 tube (Sigma Aldrich), centrifuged at 4000g for 40 minutes. Concentrated supernatants were precipitated with four volumes of ice cold acetone for two hours at -20°C. Next, the samples were centrifuged for ten minutes at 13000g followed by washing with 500 μl of ice cold acetone. The pellets were then dissolved in 100 μl of lysis buffer (1% SDS, 50 mM HEPES pH 7.6, and 1 mM DTT) and the total protein amount was estimated by DC Protein Assay (Bio-Rad). Protein digestion was performed using a modified SP3-protocol, as previously described [22]. Briefly, 90 μg of each sample was reduced with 1 mM DTT and alkylated with 4 mM Chloroacetamide. 10 μl Sera‐Mag SP3 bead mix was transferred into the protein sample together with 100% acetonitrile to a final concentration of 70%. The mix was then incubated under rotation at room temperature for 18 minutes and placed on the magnetic rack. The supernatant was discarded and the samples were washed twice with 70% ethanol and once with 100% acetonitrile. The beads-protein mixture was reconstituted in 100 μl LysC buffer (0.5 M Urea, 50 mM HEPES pH 7.6, and 1:50 enzyme (LysC) to protein ratio) and incubated overnight. Finally, trypsin was added in 1:50 enzyme to protein ratio in 100 μl 50 mM HEPES pH 7.6 and incubated overnight. The peptides were eluted from the mixture after placing the mixture on a magnetic rack, followed by peptide concentration measurement by DC Protein Assay (Bio-Rad). Samples were cleaned up by solid phase extraction (SPE strata-X-C, Phenomenex) and dried in a SpeedVac (Thermo Fisher). An aliquot of 10 μg peptides was suspended in LC mobile phase A and 1 μg peptides was injected on the LC-MS/MS system.

### LC-MS/MS analysis

Online LC-MS was performed using a Dionex UltiMate^™^ 3000 RSLCnano System coupled to a Q-Exactive mass spectrometer (Thermo Scientific). Samples were trapped on a C18 guard desalting column (Acclaim PepMap 100, 75μm x 2 cm, nanoViper, C18, 5 μm, 100 Å), and separated on a 50 cm long C18 column (Easy spray PepMap RSLC, C18, 2 μm, 100Å, 75 μmx15cm). The nano capillary solvent A consisted of 95% water, 5% DMSO, and 0.1% formic acid; and the solvent B consisted of 5% water, 5% DMSO, 95% acetonitrile, 0.1% formic acid. At a constant flow of 0.25 μl/minute, the curved gradient went from 2%B up to 40%B in 180 minutes, followed by a steep increase to 100%B in five minutes.

FTMS master scanned with 70000 resolution and mass range 300–1700 m/z were followed by data-dependent MS/MS 35000 resolution on the top 5 ions using higher energy collision dissociation (HCD) at 30–40% normalized collision energy. Precursors were isolated with a 2m/z window. Automatic gain control (AGC) targets were 1e6 for MS1 and 1e5 for MS2. Maximum injection times were 100 ms for MS1 and 150–200 ms for MS2. The entire duty cycle lasted ~2.5s. Dynamic exclusion was used with 60 second duration. Precursors with unassigned charge state or charge state 1 were excluded. An under fill ratio of 1% was used.

### Peptide and protein identification

The MS raw files were searched using Sequest-Percolator or Target Decoy PSM Validator under the software platform Proteome Discoverer 1.4 (Thermo Scientific) against human Uniprot database from March 17^th^, 2016 and filtered to a 1% FDR cut-off.

We used a precursor ion mass tolerance of 10 ppm, and product ion mass tolerances of 0.02 Da for HCD-FTMS and 0.8 Da for CID-ITMS. The algorithm considered tryptic peptides with maximum 2 missed cleavage: carbamidomethylation (C) as fixed modifications and oxidation (M) as variable modifications.

### Network analysis of the proteomic data

Proteins categorized under “immune system process” on Gene Ontology (GO) term were selected. Known protein-protein interactions from the STRING database were used to produce a network graph using igraph [23] in R v.R-3.4.2. The size represented the differential relative expression between RT4 and 5637 proteomic components and the color indicators represented betweenness (blue = low, yellow = average and red = high), as an indication of the influence of the protein in the network. Davidson Harel algorithm was used for the layout.

### Validation of proteomic analysis

Proteomic analysis of the UBC cell line supernatants was validated *in vitro*. CD8^+^ T cells from PB of healthy donors were sorted and cultured in a plate coated with 2.5 μg/ml ICAM-1 Fc chimera (Biolegend), with 10 ng/ml soluble TGFβ2 (R&D systems) and 1 μg/ml anti-CD3 stimulating antibody (clone OKT3; Biolegend). The culture was done in AIM-V medium. Cells were incubated at 37°C and 5% CO_2_ for five days and harvested for analysis using flow cytometry and RT-qPCR.

### Statistical analysis

**Fig 1:**
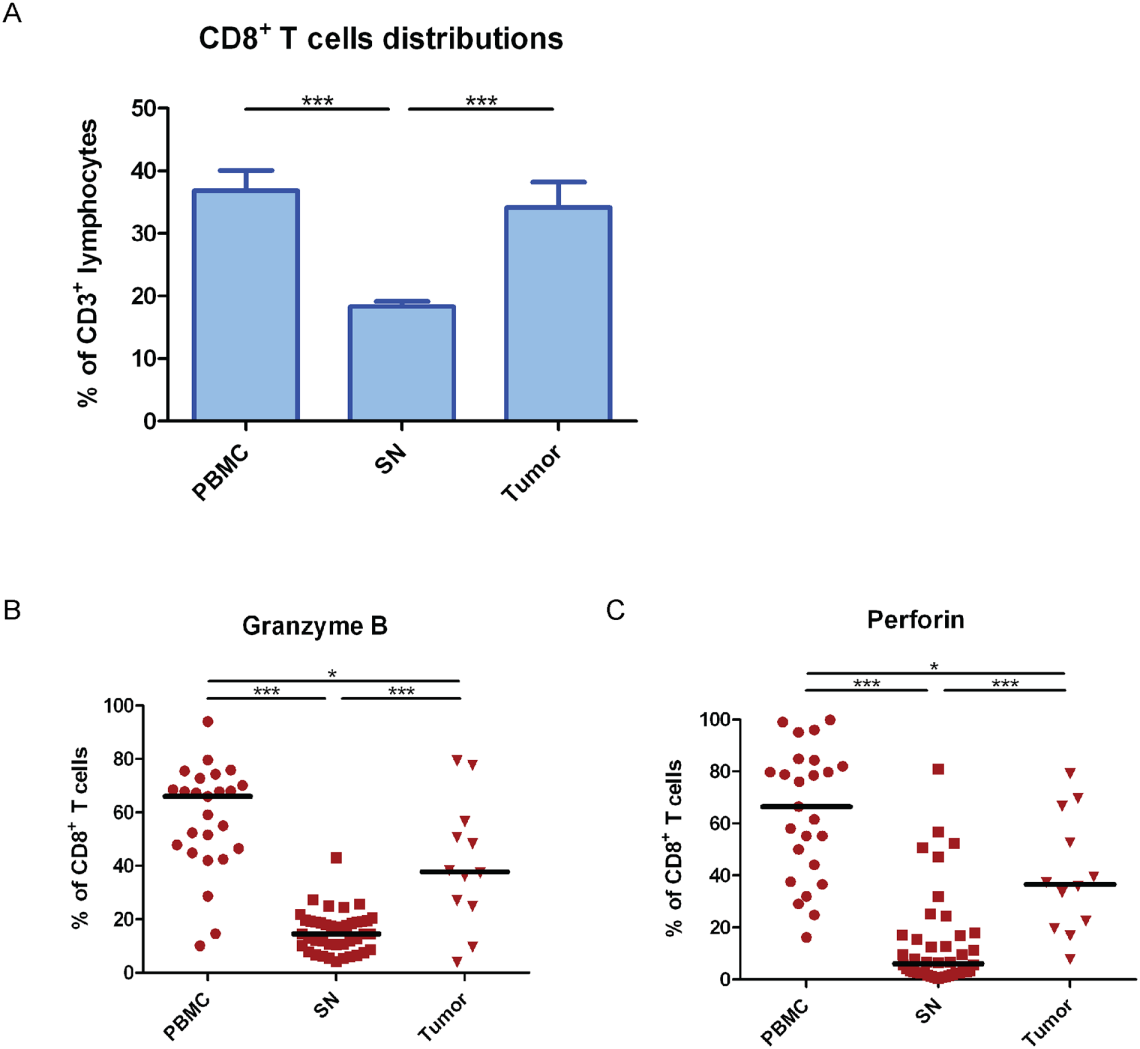
Tumor adjacent lymph nodes had lower CD8^+^ T cells cytotoxicity. Patients’ samples were received from trans-urethral resection of the bladder (TUR-B) or cystectomies (n = 27). (**A**) Comparison of CD8^+^ T cells distribution was done between peripheral blood mononuclear cells (PBMC), sentinel nodes (SN), and tumor after immunophenotyping by flow cytometry. CD8^+^ T cells distribution was calculated from CD3^+^ lymphocytes population. The data are means with the error bars indicating SEM. One-way ANOVA was used as the statistical test. (**B**) The cytotoxic phenotype of CD8^+^ T cells in different tissues from UBC patients was analyzed by flow cytometry. The frequencies of granzyme B expressing CD8^+^ T cells were compared among PBMC, SN, and tumor. (**C**) Same as in (B) but the analysis was done on perforin expressing CD8^+^ T cells. The black middle lines indicate median. Kruskal-Wallis was used as the statistical test. * p<0.05, **p<0.01, ***p<0.001, ****p<0.0001.

For the comparisons of two independent groups involving continuous dependent variables and categorical independent variables, two-sided Mann-Whitney test was used on non-parametric data. Meanwhile, Kruskal-Wallis test was used when comparing more than two independent groups with non-parametric data. For CD8^+^ T cells distribution graph (Fig 1A), one-way analysis of variance (ANOVA) test was used, assuming the normal distribution of the data. For comparison of *PRF1* gene expression and the frequency of granzyme B^+^/perforin^-^ expressing cells between different culture conditions, paired-t-test was used to compare the two dependent groups. One-way repeated-measures ANOVA was used to compare three dependent groups. Normality test was done by Kolmogorov-Smirnov test on all the data in this study. SPSS v.23 software (IBM) was used for the entire statistical test in this study.

## Results

### Decreased cytotoxicity of CD8^+^ T cells in tumor adjacent lymph nodes

Flow cytometry analysis of isolated lymphocytes from PBMC, sentinel nodes (SN), and tumor demonstrated a significantly decreased frequency of CD8^+^ T cells in SN (p<0.001), compared to PBMC and tumor (Fig 1A). However, the frequency of CD8^+^ T cells derived from PBMC was not significantly different from the frequency found in tumor (p = 0.783) (Fig 1A). Moreover, the dominant subset of CD8^+^ T cells in SN was demonstrated to be effector memory T (T_EM_) cells (CD45RA^-^ CCR7^-^) (>45%) (S1 Fig).

To characterize the cytotoxicity of CD8^+^ T cells which were dominantly T_EM_ cells, we analyzed intracellular expression of granzyme B and perforin in CD8^+^ T cells from different tissues of UBC patients (n = 27) by flow cytometry. We found that the frequency of granzyme B-expressing CD8^+^ T cells was significantly lower in SN (p<0.001) when compared to either PBMC or tumor (Fig 1B). In addition, the fraction of granzyme B-expressing CD8^+^ T cells was significantly lower in tumor compared to PBMC (p = 0.038) (Fig 1B). When we investigated the perforin expression in CD8^+^ T cells, we found that SN had a significantly decreased fraction of perforin-expressing CD8^+^ T cells than PBMC (p<0.001) (Fig 1C). The fraction of perforin-expressing CD8^+^ T cells in tumor displayed an intermediate position with significantly increased fraction of perforin-expressing CD8^+^ T cells compared to lymph nodes (p<0.001), but significantly decreased fraction compared to PBMC (p = 0.011) (Fig 1C).

### CD8^+^ T cells from sentinel lymph nodes are perforin deficient

**Fig 2:**
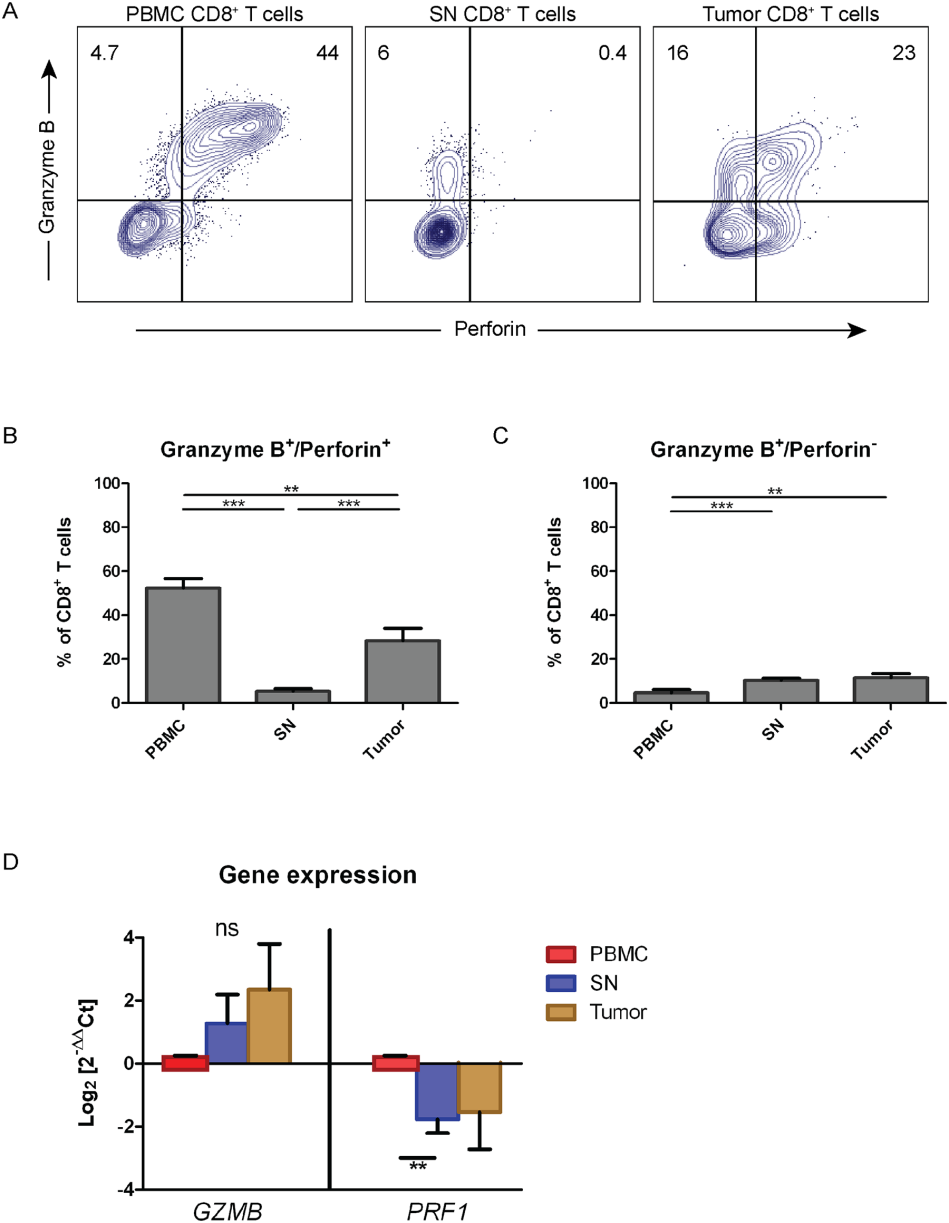
Perforin deficiency in CD8^+^ T cells from sentinel nodes. (**A**) The expression of granzyme B and perforin in CD8^+^ T cells of different tissues were phenotyped by flow cytometry. The co-expression pattern in CD8^+^ T cells was shown in dot plots and gated for distinguishing between double and single expression of granzyme B and perforin. The gate was based on isotype control and the frequency of granzyme B and perforin expression was counted out of CD8^+^ T cells. Dot plots showed a representative data from a patient underwent transurethral resection of the bladder (TUR-B) and cystectomy. (**B**) The frequency of granzyme B^+^/perforin^+^ CD8^+^ T cells from PBMC, SN, and tumor tissues was shown in graphs and was calculated out of CD8^+^ T cells (n = 27). (**C**) Same as in (B) but the analysis was done on granzyme B^+^/perforin^-^ CD8^+^ T cells. The data are means with the error bars indicating SEM. Kruskal-Wallis was used as the statistical test. (**D**) The expression of gene responsible in encoding granzyme B (*GZMB*) and perforin (*PRF1*) in CD8^+^ T cells isolated from PBMC, SN, and tumor (n = 6). RT-qPCR was done to analyze the gene expression followed by quantification using 2^-ΔΔCt^ method. The fold change was calculated in regards of PBMC as control, with *RPII* gene used as the housekeeping gene. The data are the means of Log_2_ of fold change (2^-ΔΔCt^) with the error bars indicating SEM. Kruskal-Wallis was used as the statistical test on each gene. *p<0.05, **p<0.01, ***p<0.001, ****p<0.0001.

Next, we investigated co-expression of granzyme B and perforin in CD8^+^ T cells. Surprisingly, we observed that CD8^+^ T cells acquired from SN displayed a perforin-deficient phenotype, with intact granzyme B expression (Fig 2A, middle panel). This phenomenon was not observed in CD8^+^ T cells from PBMC. CD8^+^ T cells from tumor displayed an intermediate phenotype with a decreased fraction of perforin-expressing cells (Fig 2A, right panel). High frequencies of granzyme B^+^/perforin^+^ CD8^+^ T cells were found in PBMC (mean = 52%) (Fig 2B). We observed a 50% decrease of granzyme B^+^/perforin^+^ of tumor-derived CD8^+^ T cells compared to PBMC (Fig 2A and 2B). In contrast, the fractions of granzyme B^+^/perforin^+^ CD8^+^ T cells in SN were below 6% (p<0.001 vs PBMC and tumor) (Fig 2B). The presence of granzyme B^+^/perforin^-^ CD8^+^ T cells demonstrated an opposite pattern among PBMC, SN, and tumor. The mean frequency of granzyme B^+^/perforin^-^ CD8^+^ T cells was significantly lower in PBMC (<5%) compared to SN and tumor (>12%) (p<0.01) (Fig 2C). However, there was no significant difference in the frequency of this subset between SN and tumor (p = 0.526).

To investigate the expression level of granzyme B (*GZMB*) and perforin (*PRF1*) at steady state, we performed RT-qPCR on sorted CD8^+^ T cells from PBMC, SN, and tumor (Fig 2D). CD8^+^ T cells from PBMC were used as comparator. Granzyme B was shown to have 1.3-fold upregulation in CD8^+^ T cells from SN and 2.4-fold increased expression in CD8^+^ T cells from tumor compared to PBMC (p = 0.125 and p = 0.526, respectively) (Fig 2D). In contrast, the *PRF1* transcript was significantly downregulated in CD8^+^ T cells from SN (1.8 fold) when compared to CD8^+^ T cells from PBMC (p = 0.002) (Fig 2D). *PRF1* expression was decreased 1.5 fold in CD8^+^ T cells from tumor compared to PBMC although not significant (p = 0.526). Thus, the decrease of perforin protein expression in CD8^+^ T cells from SN and tumor (Fig 2A, 2B and 2C) was reflected in and supported by a decreased expression of the perforin coding gene (Fig 2D).

### Perforin production and secretion are low even after in vitro reactivation of tumor reactive CD8^+^ T cells

**Fig 3:**
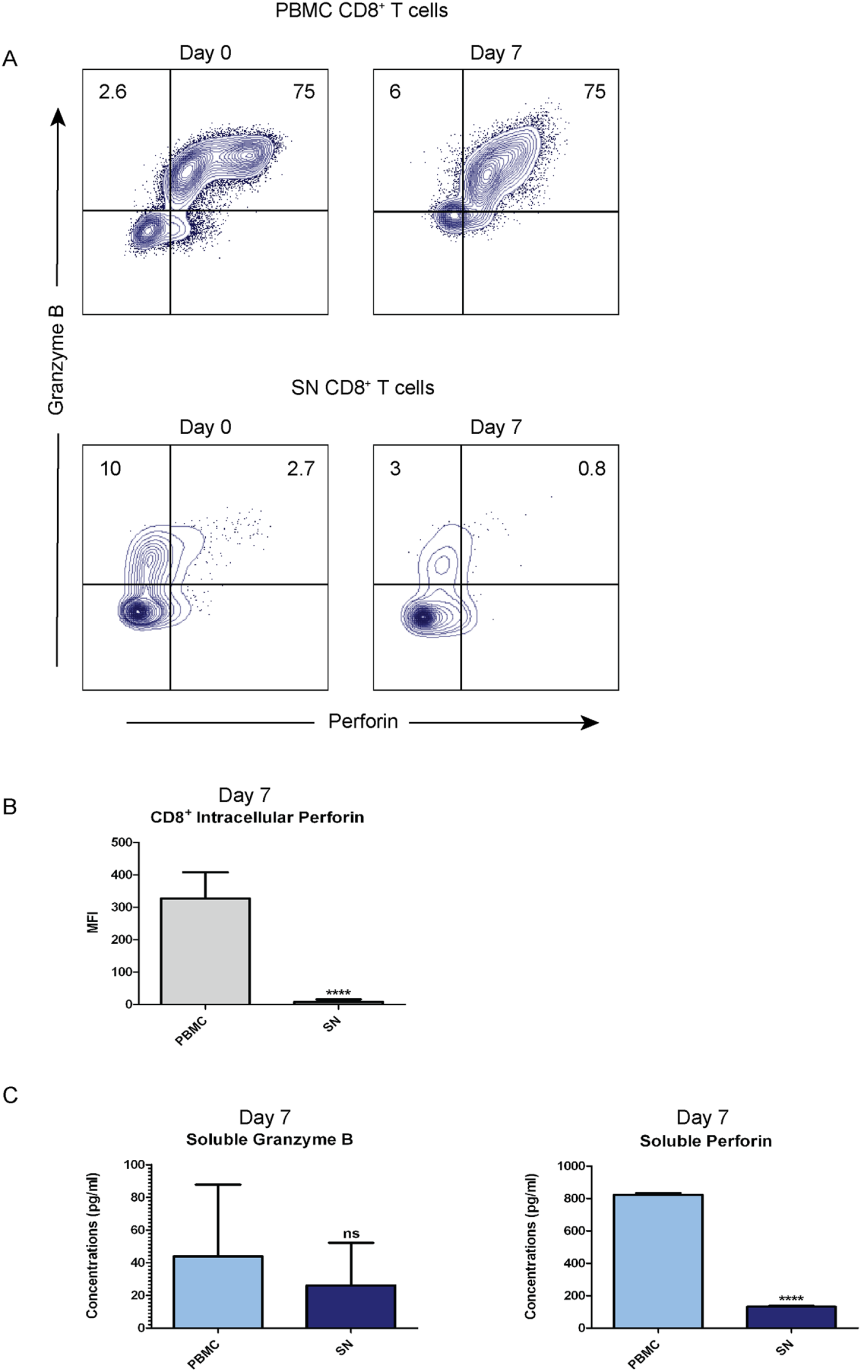
Production and secretion of perforin in SN CD8^+^ T cells are low after *in vitro* reactivation. Lymphocytes isolated from peripheral blood (PBMC) and sentinel node (SN) were cultured for seven days with addition of autologous tumor homogenate. (**A**) Flow cytometry was done to phenotype the co-expression in CD8^+^ T cells from PBMC and SN before and after reactivation. The results were shown in dot plots and gated based on isotype control. The frequency of granzyme B and perforin expression was counted out of CD8^+^ T cells. Dot plots showed data from a representative cystectomized patient. (**B**) Intracellular perforin was measured by Median Fluorescence Intensity (MFI) post 7-day culture using flow cytometry from (A). The data are means with error bars indicating SEM. Mann-Whitney was used as the statistical test. (**C**) The concentrations (pg/ml) of secreted granzyme B and perforin after seven days of culture were analyzed by ELISA and compared between *in vitro* culture supernatants of PBMC and SN. The data are means with error bars indicating SEM. Mann-Whitney was used as the statistical test. *p<0.05, **p<0.01, ***p<0.001, ****p<0.0001.

In order to explore whether perforin expression can be restored from tumor reactive CD8^+^ T cells, we cultured T cells from PBMC and SN with autologous tumor homogenate as antigen for seven days. The majority of CD8^+^ T cells from PBMC were granzyme B^+^/perforin^+^ double positive (75%) both at baseline and post-culture (Fig 3A, top panels). However, SN culture contained few granzyme B^+^/perforin^+^ double positive CD8^+^ T cells (<3%) at baseline and they decreased further during the culture period (0.8%) (Fig 3A, bottom panels). Low intracellular perforin in CD8^+^ T cells from SN compared to PBMC (p<0.0001) (Fig 3B) indicated low perforin production in SN post reactivation. Furthermore, we investigated the concentrations of secreted soluble granzyme B and perforin in the culture supernatant by ELISA after seven days of tumor antigen activation. We found no significant difference in granzyme B secretion between PBMC and SN lymphocytes (p = 0.683) (Fig 3C, left panel). However, the perforin secretion was significantly higher from PBMC lymphocytes (>800pg/ml) compared to SN lymphocytes (<150pg/ml) (p<0.0001) (Fig 3C, right panel).

### Perforin-deficient CD8^+^ T cells in sentinel nodes display T_EM_ cells with exhausted Tc2 anti-inflammatory phenotype

**Fig 4:**
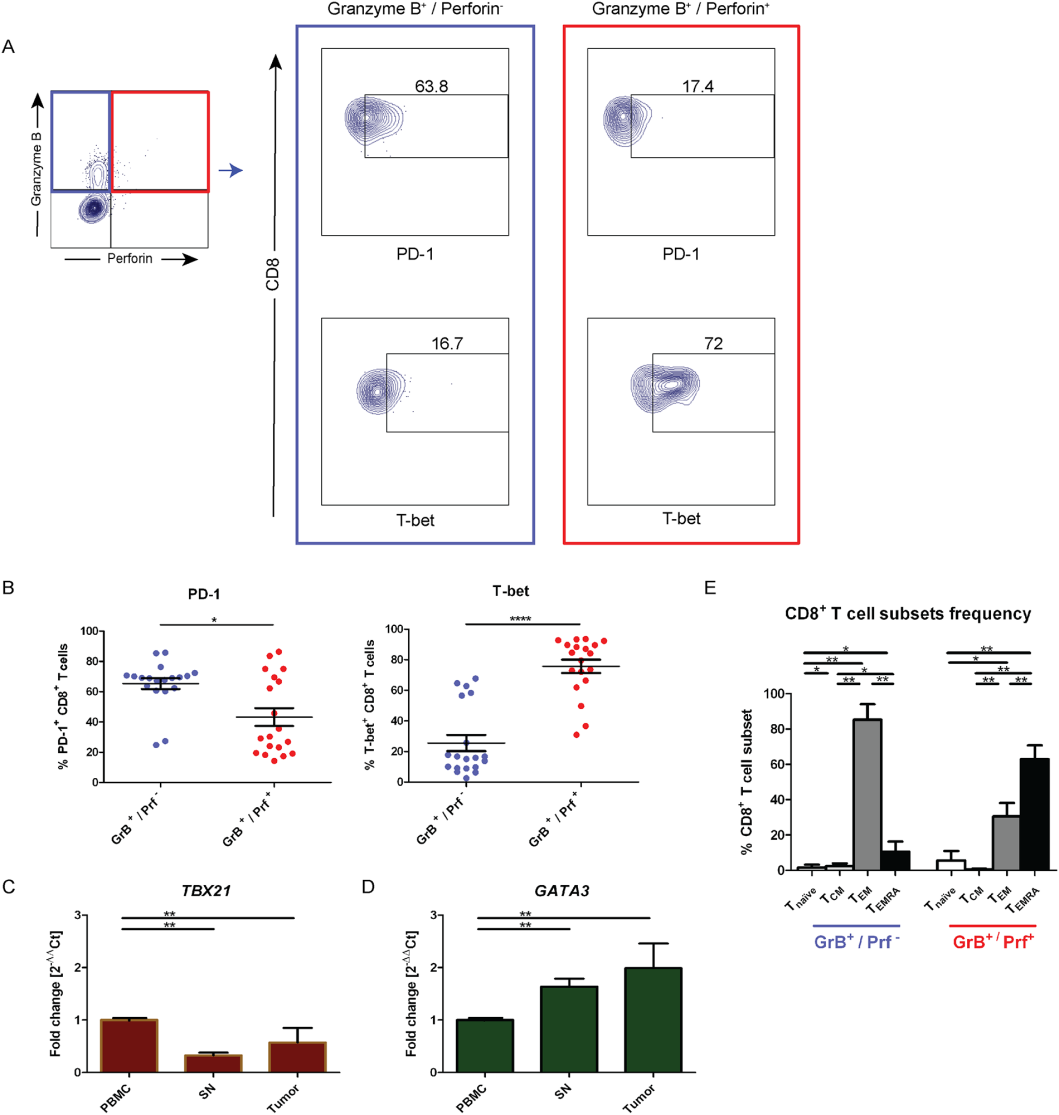
Sentinel node CD8^+^ T cells with perforin deficiency are exhausted Tc2 cells. (**A**) CD8^+^ T cells isolated from sentinel node (SN) were further phenotyped using flow cytometry to demonstrate the difference in T cells exhaustion markers expression (PD-1) and Tc1 transcription factor (T-bet) between granzyme B^+^/perforin^−^CD8^+^ T cells (green box) and granzyme B^+^/perforin^+^ CD8^+^ T cells (red box). The expression of PD-1 and T-bet were shown in dot plots from a representative patient and gated based on isotype control. (**B**) The frequency of PD-1 and T-bet from (A) was calculated either out of granzyme B^+^/perforin^−^or granzyme B^+^/perforin^+^ CD8^+^ T cells. The data are means with the error bars indicating SEM. Mann-Whitney was used as the statistical test. (**C**) The expression of T-bet, encoded by *TBX21* gene, was compared among CD8^+^ T cells sorted from peripheral blood mononuclear cells (PBMC), sentinel node (SN), and tumor. mRNA was extracted from the sorted cells and the *TBX21* gene expression was analyzed by RT-qPCR. The expression of *TBX21* was quantified using 2^-ΔΔCt^ method and the fold change was calculated in regards of PBMC as control. *RPII* gene was used as housekeeping gene. The data are means with error bars indicating SEM. Kruskal-Wallis was used as the statistical test. (**D**) Same as in (C), but the analysis was done on *GATA3* gene expression. (**E**) The frequency of naïve T cells (CD45RA^+^ CCR7^+^), central memory T (T_CM_) cells (CD45RA^-^ CCR7^+^), effector memory T (T_EM_) cells (CD45RA^-^ CCR7^-^), and effector memory T with CD45RA expression (T_EMRA_) cells (CD45RA^+^ CCR7^-^) was calculated either out of granzyme B^+^/perforin^−^or granzyme B^+^/perforin^+^ CD8^+^ T cells. The data are means with the error bars indicating SEM. Kruskal-Wallis was used as the statistical test. * p<0.05, **p<0.01, ***p<0.001, ****p<0.0001.

In order to ascertain whether low expression of perforin in CD8^+^ T cells was associated with cell exhaustion, we analyzed PD-1 expression by flow cytometry. Perforin-deficient CD8^+^ T cells displayed an increased expression level of PD-1 and in addition demonstrated a significantly higher fraction of PD-1 than granzyme B^+^/perforin^+^ CD8^+^ T cells (p = 0.017) (Fig 4A and 4B, left panel). This suggests that perforin deficient CD8^+^ T cells from the SN are exhausted.

To further dissect perforin deficiency in CD8^+^ T cells from SN, we analyzed the expression of the Tc1 transcription factor, T-bet [24]. Granzyme B^+^/perforin^-^ CD8^+^ T cells were shown to possess significantly lower T-bet expression when compared to granzyme B^+^/perforin^+^ CD8^+^ T cells (p<0.0001) (Fig 4A and 4B, right panel).

We next analyzed the mRNA expression of the hallmark transcription factors for Tc1 (*TBX21*) and Tc2 (*GATA3*) in sorted CD8^+^ T cells from PBMC, SN, and tumor. The *TBX21* transcript was significantly downregulated (3 fold) in SN CD8^+^ T cells compared to PBMC (p<0.01) and in the CD8^+^ T cells from the tumor (p<0.01) (Fig 4C). No significant difference between *TBX21* expression in SN and tumor CD8^+^ T cells was found (p = 0.513). When analyzing the Tc2 transcription factor, GATA-3, we found 1.6 times upregulation in CD8^+^ T cells derived from SN (p<0.01) and 2 times upregulation in CD8^+^ T cells from tumor when compared to from PBMC (p<0.01) (Fig 4D). We found no significant difference between *GATA3* expression between SN-derived and tumor-derived CD8^+^ T cells (p = 0.827). Additionally, granzyme B^+^/perforin^-^ CD8^+^ T cells from SN were 85% T_EM_ cells (Fig 4E). Taken together, perforin-deficient CD8^+^ T cells in SN were T_EM_ cells with accumulated PD-1 and GATA-3 expression, suggesting an induced exhausted anti-inflammatory phenotype.

### Perforin expression in CD8^+^ T cells from SN can be rescued by providing Tc1 conditions

**Fig 5:**
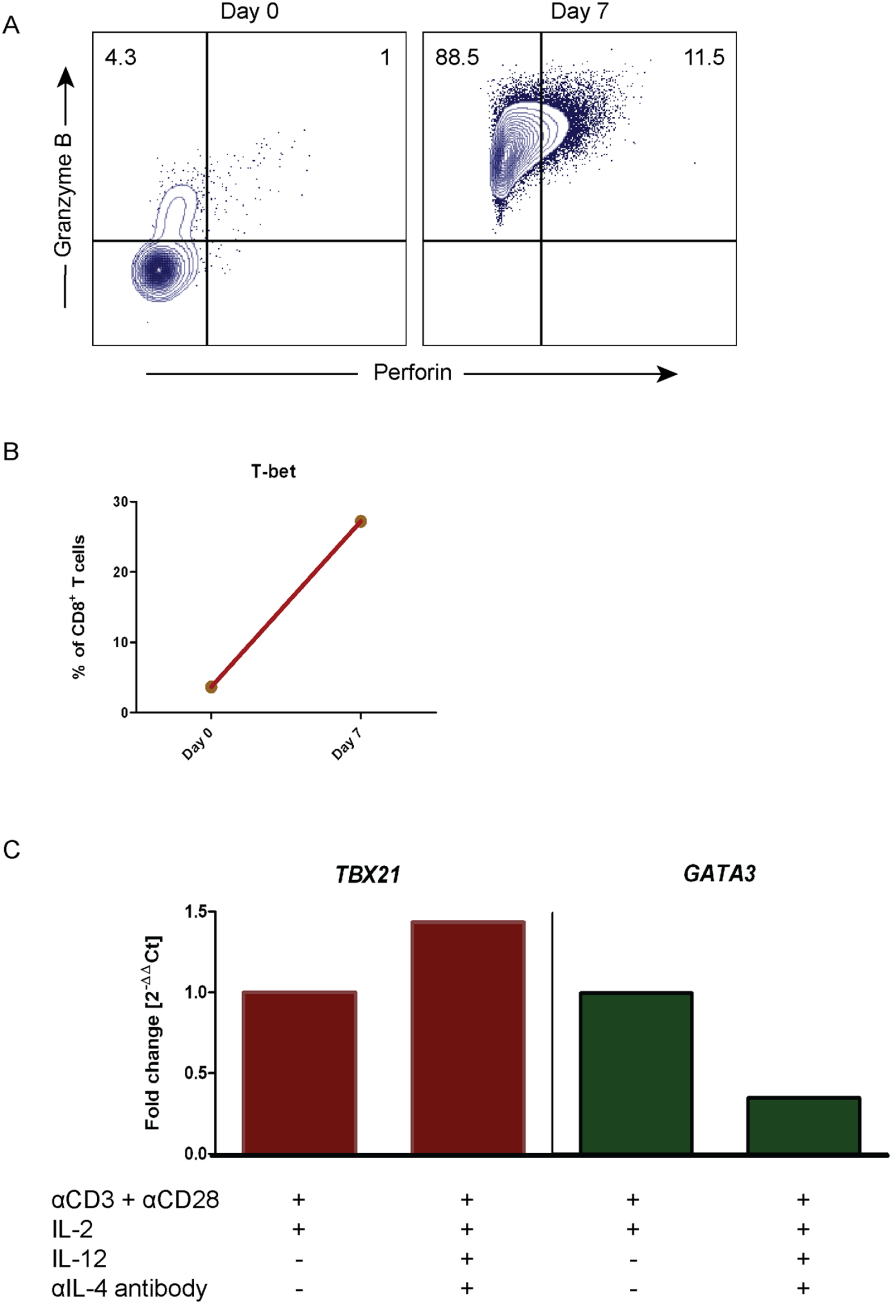
Tc1 conditions can restore perforin expression in CD8^+^ T cells from sentinel nodes. CD8^+^ T cells sorted from sentinel node (SN) were cultured in Tc1 conditions *in vitro* for seven days in order to rescue perforin expression. These SN-derived CD8^+^ T cells were stimulated with anti-CD3 and anti-CD28 stimulating antibodies with the presence of IL-12 and IL-2 cytokines, as well as an anti-IL-4 neutralizing antibody. At the end of the culture, cells were analyzed by flow cytometry and RT-qPCR. (**A**) Dot plots showed the flow cytometry data from a representative patient for granzyme B vs. perforin expression, before and after the stimulation. The gate was based on isotype control and the frequency of granzyme B and perforin expression was counted out of CD8^+^ T cells. (**B**) Flow cytometry result of T-bet expression percentage from CD8^+^ T cells pre- and post-stimulation was analyzed. The frequency of T-bet expression was calculated from CD8^+^ T cells. (**C**) *TBX21* and *GATA3* gene expression analysis was done by RT-qPCR from cells in different culture conditions. *RPII* gene was used as housekeeping gene and the fold change was calculated based on cells without IL-12 and anti-IL-4 as control using 2^-ΔΔCt^ method.

Next, we attempted to restore perforin expression in CD8^+^ T cells by providing a Tc1 culture condition. SN-derived CD8^+^ T cells were cultured *in vitro* in the presence of recombinant human IL-12, IL-2 cytokines, and anti-IL-4 neutralizing antibody, in the presence of activating anti-CD3 and anti-CD28 antibodies for seven days. The frequency of granzyme B^+^/perforin^+^ CD8^+^ T cells increased from 1% to 11.5% in the Tc1-promoting culture (Fig 5A), indicating that perforin synthesis was induced. In addition, T-bet expression in CD8^+^ T cells increased 7.5 times after seven days of Tc1-promoting conditions (Fig 5B). Further analysis demonstrated that the *TBX21* gene expression upregulation was dependent on Tc1 conditions i.e. IL-12 and anti-IL-4 (Fig 5C, left panel). Accordingly, the *GATA3* transcript was downregulated in the Tc1-promoting environment (Fig 5C, right panel). Thus, perforin expression and Tc1 commitment in CD8^+^ T cells from SN are rescued by providing Tc1-promoting conditions.

### Muscle invasive UBC induced perforin deficiency through ICAM-1 and TGFβ2 signaling

**Fig 6:**
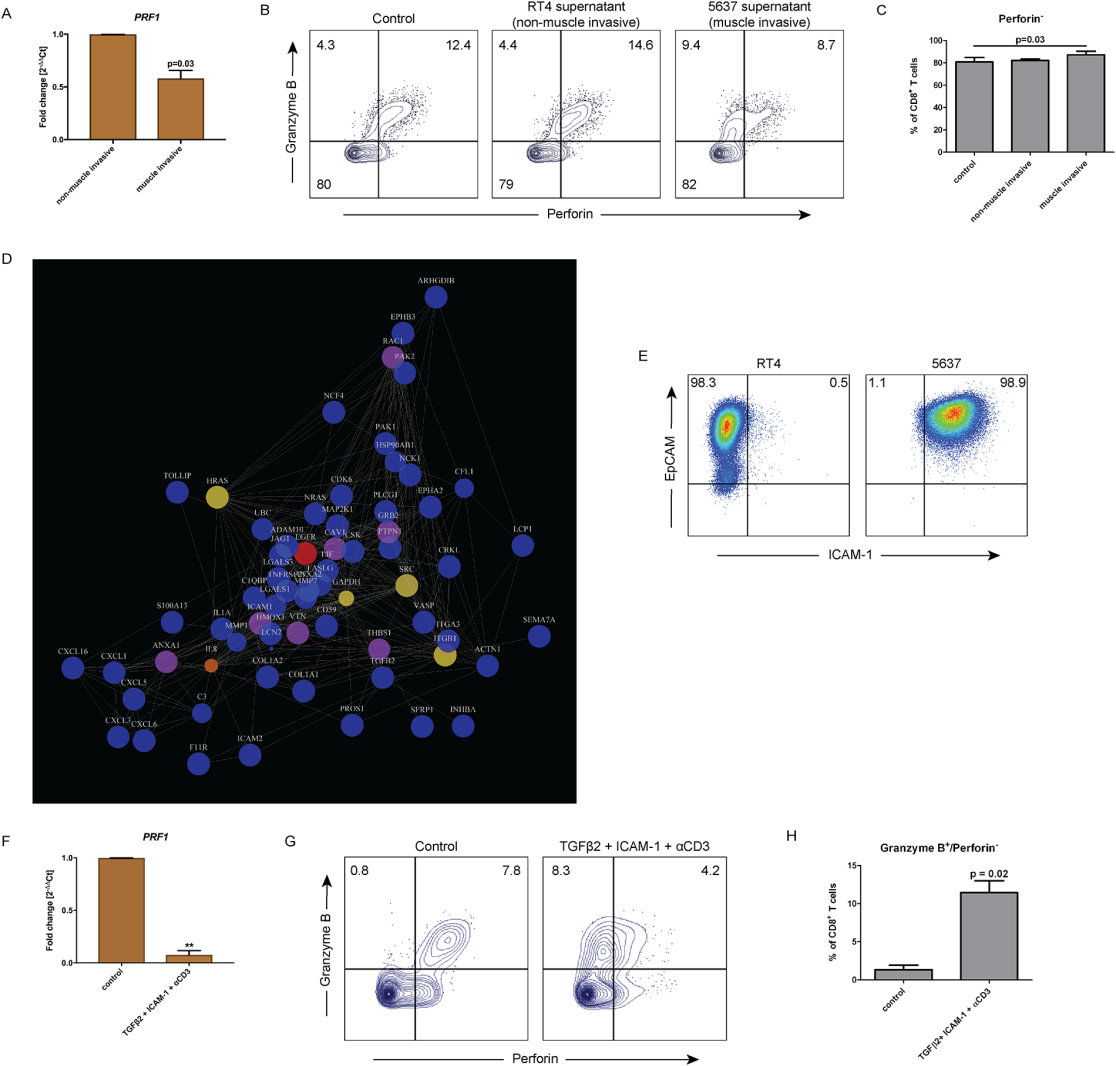
ICAM-1 and TGFβ2 signal from muscle invasive UBC causes perforin downregulation. Culture supernatants of urothelial bladder cancer (UBC) cell lines were acquired from RT4 (non-muscle invasive) and 5637 (muscle invasive) cell lines. CD8^+^ T cells were then isolated from peripheral blood of healthy donor and cultured in *vitro* with these supernatants for five days. (**A**) Analysis of perforin coding gene (*PRF1*) expression was done by RT-qPCR. mRNA was extracted post-culture from the cells of the culture groups. Bar graphs show different expression of *PRF1* in CD8^+^ T cells cultured *in vitro* between RT4 (non-muscle invasive) and 5637 (muscle invasive) supernatant. *RPII* gene was used as housekeeping gene and the fold change was calculated in regards of RT4 medium using 2^-ΔΔCt^ method. The data are means with error bars indicating SEM. Paired-t-test was used as the statistical test. (**B**) Flow cytometry analysis of CD8^+^ T cells at the end of culture was done. The results comparing three groups were shown in dot plots from a representative healthy donor and gated based on isotype control. (**C**) The frequency of perforin^-^ CD8^+^ T cells from (B) was counted out of CD8^+^ T cells. The data are means with the error bars indicating SEM. One-way repeated-measure ANOVA was used as the statistical test. (**D**) Mass spectometry (MS) analysis identified proteins expressed by RT4 and 5637 cell line. Proteins under the category “immune system process” on the GO (Gene Ontology) term were selected for network analysis based on STRING database. Size represented differential expression between RT4 and 5637 supernatants and the color represented betweenness which marked the influence of the protein to the network. Color indicators: blue = low, yellow = average and red = high. (**E**) The expression of ICAM-1 was validated by flow cytometry on RT4 and 5637 cell line. RT4 and 5637 cells were identified by EpCAM expression. (**F**) Validation of perforin downregulation by ICAM-1 and TGFβ2 was done *in vitro* on CD8^+^ T cells isolated from healthy donors in the presence of anti-CD3 stimulating antibody for 5 days. Perforin coding gene (*PRF1*) expression was done by RT-qPCR. mRNA was extracted post-culture from the cells. Bar graphs show different expression of *PRF1* in CD8^+^ T cells cultured *in vitro* between control and TGFβ2 + ICAM-1 + αCD3. RPII gene was used as housekeeping gene and the fold change was calculated in regards of blank medium using 2-ΔΔCt method. The data are means with error bars indicating SEM. Paired-t-test was used as the statistical test. (**G**) Flow cytometry analysis of CD8^+^ T cells at the end of culture was done. The results were shown in dot plots and gated based on isotype control from a representative healthy donor. The frequency of granzyme B and perforin expression was counted out of CD8^+^ T cells. (**H**) The frequency of granzyme B^+^/perforin^-^ expressing cells from (G) was counted out of CD8^+^ T cells. The data are means with error bars indicating SEM. Paired-t-test was used as the statistical test. * p<0.05, **p<0.01, ***p<0.001, ****p<0.0001.

In order to explore the possible tumor immune escape mechanism that caused perforin deficiency, we did an *in vitro* culture of sorted CD8^+^ T cells from PB of healthy donors in the presence of culture supernatants from the UBC cell lines: RT4 (non-muscle invasive) and 5637 (muscle invasive) for five days. We observed that *PRF1* gene expression was significantly lower (p = 0.03) in CD8^+^ T cells cultured with the supernatant from muscle invasive 5637 UBC cell line compared to post-culture in the non-muscle invasive RT4 supernatant (Fig 6A). We showed that the frequency of granzyme B^+^/perforin^+^ CD8^+^ T cells significantly reduced and shifted into granzyme B^+^/perforin^-^ CD8^+^ T cells after cultured in supernatant from the muscle invasive 5637 cell line (p = 0.03) (Fig 6B and 6C). Therefore, we hypothesized that a soluble protein is secreted by the muscle invasive UBC tumor cells which induced downregulation of perforin expression as a part of the immune escape mechanism.

Conducting a proteomic analysis of RT4 and 5637 cell culture supernatants by mass spectrometry, we selected proteins from both supernatants that fell into the category of “immune system process” under the GO (Gene Ontology) term. Network analysis was done on the selected proteins using the STRING database (Fig 6D). We found that ICAM-1 and TGFβ2 were only expressed in the 5637 supernatant (shown by bigger sizes in the plot). In addition, proteins involved in tumor cells invasion such as CXCL1, CXCL5, and MMP1 were displayed to be expressed and interacted with ICAM-1 and TGFβ2 in the network analysis (Fig 6D).

The expression of ICAM-1 was validated to be expressed on the cell surface of 5637 cell line (98.9% positive cells) but not on the RT4 cell surface (Fig 6E). Next, we validated the capability of ICAM-1 and TGFβ2 to suppress perforin expression. Sorted CD8^+^ T cells from PB of healthy donors were cultured *in vitro* on a plate coated with ICAM-1 Fc chimera to mimic the engagement of ICAM-1-mediated engagement of tumor cells binding to LFA-1 on the surface of CD8^+^ T cells, in the presence of soluble human recombinant TGFβ2 and anti-CD3. The *PRF1* transcript was significantly downregulated (p = 0.0019) after five days of culture with TGFβ2, ICAM-1, and anti-CD3 compared to control (Fig 6F). Additionally, the frequency of granzyme B^+^/perforin^-^ CD8^+^ T cells increased 10 fold after treatment with TGFβ2, ICAM-1, and anti-CD3 compared to control (p = 0.02) (Fig 6G and 6H). This was consistent with the suppression of perforin effect exerted by the muscle invasive UBC cell line culture supernatant (Fig 6A, 6B and 6C). We noticed that the exhaustion marker PD-1 expression was not different after stimulation with TGFβ2, ICAM-1, and anti-CD3 compared to when ICAM-1 stimulation was eliminated (TGFβ2 and anti-CD3 alone) (S2 Fig). This suggested that TGFβ2 alone is enough to regulate CD8^+^ T cell exhaustion. However, in order to downregulate perforin, TGFβ2 and ICAM-1 stimulation are necessary in combination.

## Discussion

To the best of our knowledge, we here for the first time describe perforin deficiency in SN-derived CD8^+^ T cells due to a tumor-induced mechanism. Our surprising discovery demonstrated that perforin in CD8^+^ T cells from sentinel nodes was almost completely missing, whereas granzyme B expression was preserved (Fig 2), suggesting an immune escape mechanism. The loss of perforin expression was displayed to be originated from the level of *PRF1* transcript in SN, which was also seen in tumor CD8^+^ T cells (Fig 2D). These findings prompted us to focus in elucidating the phenotype of SN CD8^+^ T cells. Accordingly, we were able to demonstrate tumor escape using supernatant from the muscle invasive UBC cell line (5637), where the supernatant contained soluble ICAM-1 and TGFβ2 that induced perforin downregulation (Fig 6). Consequently, short time culture for seven days with activating signal 1 and 2 in Tc1 promoting conditions rescued perforin-deficient cells (Fig 5).

Perforin is an important cytotoxic constituent of CD8^+^ T cells which acts as a weapon to kill tumor cells. Accordingly, mutation in the perforin coding gene (*PRF1*) increases the susceptibility to develop cancers [25]. Previously it was reported that perforin transcript is downregulated in TIL from patients with lung adenocarcinoma [26]. Moreover, in the context of infection, perforin deficiency in granzyme-positive CD8^+^ T cells from lymph nodes of acute and chronic HIV infections has been demonstrated [27, 28].

In cancer, SN receive higher lymphatic flow from the tumor due to higher interstitial fluid pressure and increased neo-lymphangiogenesis [20]. This increases the reception of tumor antigens and cytokines into the SN. Furthermore, as declared in the concept of immunoediting, the tumor cells are capable to escape destruction by immune cells [29]. Therefore, SN may represent the primary tumor microenvironment and be modified to be tolerogenic as an escape mechanism [30], supporting our findings.

We noted elevated levels of PD-1 expression on perforin-deficient CD8^+^ T cells in SN, with concomitant low T-bet expression (Fig 4A and 4B), suggesting exhaustion. Exhaustion of tumor specific CD8^+^ T cells is caused by inflammation formed by the tumor microenvironment, resulting in chronic antigenic exposure towards CD8^+^ T cells [31, 32]. Exhausted CD8^+^ T cells exhibit impairment in proliferation, cytokine production, and cytotoxicity. Several mechanisms behind exhaustion-driven immune impairment by PD-1 have been explained, including inhibition of ZAP70, CD3ζ, and co-stimulatory receptor CD28 phosporylation [33, 34]. In addition, metabolic defects in glucose metabolism due to mitochondrial insufficiency [35, 36] and modulation of phosphoinositide 3-kinase (PI3K), AKT, and RAS pathways have been demonstrated as the results of exhaustion [37]. Interestingly, these pathways may inhibit the transcription of T-bet, responsible in the effector cell formation [38]. Furthermore, glycogen synthase kinase 3 (GSK-3) inactivation in a mouse model, causes inhibition of PD-1 transcription and enhancement of T-bet expression which subsequently restore CD8^+^ T cell cytotoxicity [39, 40]. Accordingly, blockade of PD-1 *in vivo* in humans by immune checkpoint blockade has an impact on improved cytotoxicity and tumor regression [41].

We found that the Tc2 transcription factor, GATA-3, was upregulated in CD8^+^ T cells from SN and tumor, with concomitant downregulation of the Tc1 transcription factor, T-bet (Fig 4C and 4D). This indicated that low perforin was the result of Tc2-polarized tumor microenvironment, as shown in human cervical cancer [42]. Moreover, low T-bet expression would be responsible for low perforin transcription as T-bet binds in the promoter region of perforin gene to initiate transcription [43]. We attempted to rescue perforin expression by providing Tc1 promoting conditions with IL-12 and anti-IL-4 which resulted in an increased expression of perforin (Fig 5). IL-12 had been reported to trigger mitochondrial function of exhausted HBV-specific CD8^+^ T cells in human [44]. This in turn would cause these cells to recover from exhaustion and gain their cytotoxicity, which was consistent to our data.

Our final finding suggested that invasive tumor cells induced perforin downregulation within SN by ICAM-1 and TGFβ2 signaling. It has been shown that ICAM-1 expressed on the tumor cells binds to LFA-1 on CD8^+^ T cells and promotes activation and cytotoxicity of CD8^+^ T cells in a TCR-dependent fashion [45]. However, the presence of TGFβ2 secreted by invasive UBC tumor cells caused CD8^+^ T cells to be incapable to produce perforin, despite the presence of signal 2 (Fig 6F, 6G and 6H). It is known that TGFβ is responsible for causing transcriptional repression of perforin within CD8^+^ T cells as a tumor immune escape mechanism [46]. Furthermore, as the tumor cells secrete TGFβ, deletion of TGFβ receptor 2 (*Tgfbr2*) within the tumor is demonstrated to increase CXCL1/CXCL5 –CXCR2 chemokine and chemokine receptor signaling. This will result in higher recruitment of myeloid-derived suppressor cells (MDSCs) to the tumor microenvironment. MDSCs will in turn produce MMPs and TGFβ that would support the invasion of the tumor cells [47]. Our network analysis supported this in which CXCL1, CXCL5, and MMP1 displayed interactions with TGFβ2 (Fig 6D).

As a novel synergistic tumor immune escape was exerted by ICAM-1 and TGFβ2 signal, it opens a new potential in translational application. Interestingly, some clinical trials have been initiated to target ICAM-1 and TGFβ2 individually as the possibilities for future cancer immunotherapies [48, 49]. This can further lead into combinations of immunotherapy as new strategies against cancer.

The limitation of this study was that only two signals out of ~20 signals (expressed only by invasive tumor cells) from the proteomic analysis was validated (ICAM-1 and TGFβ2). However, using TGFβ2 and ICAM-1, we could recapitulate the immunosuppressive microenvironment of the tumor, diminishing 50% of perforin expression on naïve CD8^+^ T cells. This suggests that the majority of the suppression can be explained and additional signals contribute to the perforin downregulation. We believe that it is the general immune escape mechanism generated by solid tumors, but at this stage, we have too few observations from other malignancies to firmly draw that conclusion.

Taken together, our data demonstrated that low perforin in CD8^+^ T cells from SN as a result of tumor immune escape mechanism in UBC patients. The tumor was capable of modifying the SN environment to be tolerogenic by forming chronic inflammation, causing the synergistic effects of ICAM-1 and TGFβ2 signal, together with exhaustion and Tc2 skewed environment, to suppress CD8^+^ T cells cytotoxicity [50]. By identifying this novel tumor immune escape mechanism, we hope that our findings could be beneficially translated into new potential immunotherapy strategies against cancer.

## Supporting information

S1 Fig The distribution of CD8^+^ T cell subsets. The frequency of naïve T cells (CD45RA^+^ CCR7^+^), central memory T (T_CM_) cells (CD45RA^-^ CCR7^+^), effector memory T (T_EM_) cells (CD45RA^-^ CCR7^-^), and effector memory T with CD45RA expression (T_EMRA_) cells (CD45RA^+^ CCR7^-^) was calculated out of CD8^+^ T cells from PBMC, SN, and tumor. The data are means with the error bars indicating SEM. Kruskal-Wallis was used as the statistical test. * p<0.05, **p<0.01, ***p<0.001, ****p<0.0001.
(TIF)

S2 Fig PD-1 expression is regulated by TGFβ2 alone. CD8^+^ T cells isolated from healthy donors were cultured *in vitro* in the presence of TGFβ2, anti-CD3 stimulating antibody, with or without ICAM-1 Fc chimera. Flow cytometry analysis of PD-1 expression on CD8^+^ T cells was performed at baseline (day 0), day 3, and day 5. The frequency of PD-1-expressing cells was counted out of CD8^+^ T cells. The data are means with error bars indicating SEM. One-way repeated-measure ANOVA was used as the statistical test. * p<0.05, **p<0.01, ***p<0.001, ****p<0.0001.
(TIF)
