# Supplementary figures and images for: Urothelial bladder cancer may suppress perforin expression in CD8+ T cells by an ICAM-1/TGFβ2 mediated pathway
Source: PLoS One. 2018 Jul 2;13(7):e0200079. doi: 10.1371/journal.pone.0200079 (PMC6028111; doi:10.1371/journal.pone.0200079)

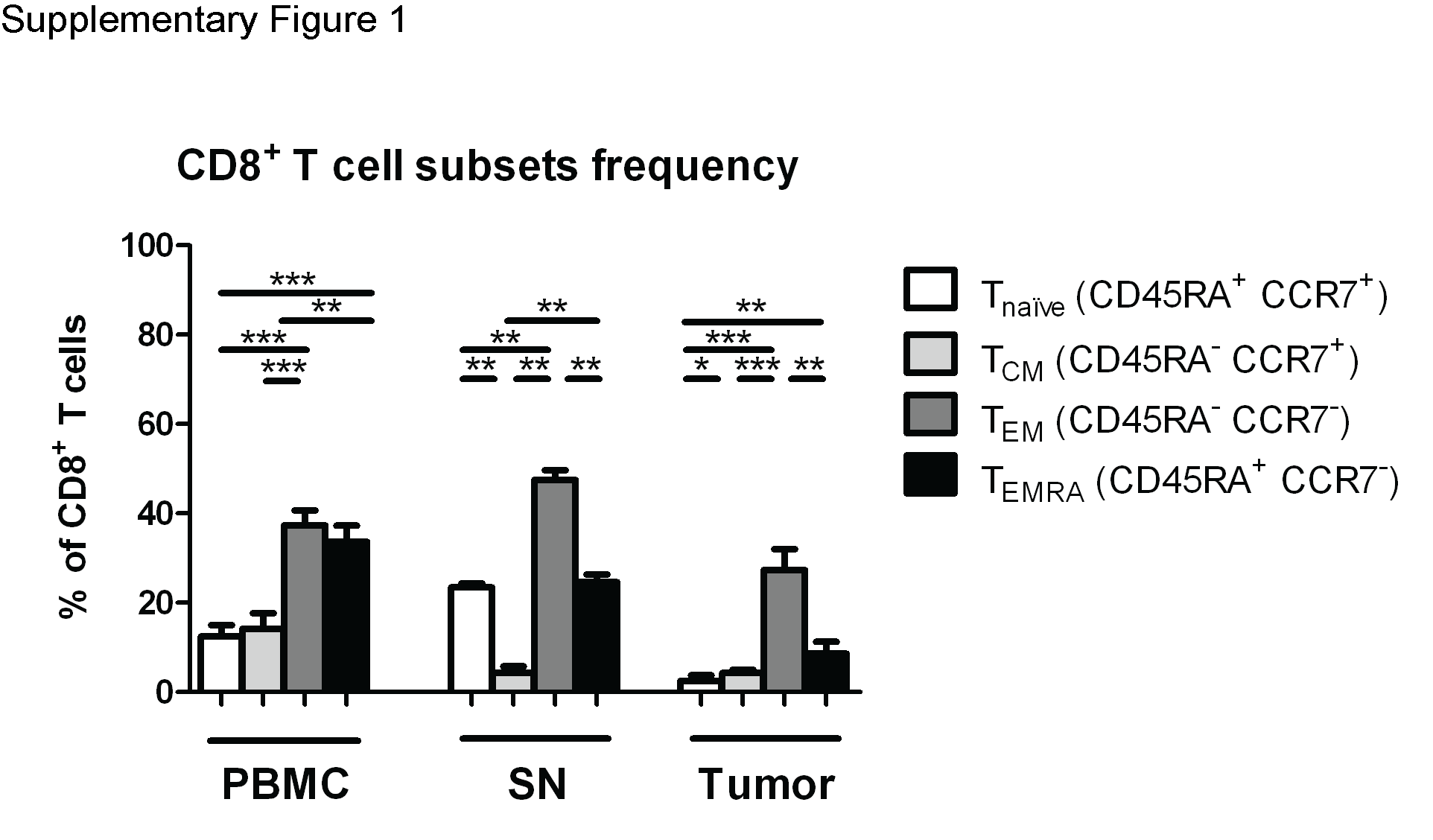

Supplement: S1 Fig — The frequency of naïve T cells (CD45RA+ CCR7+), central memory T (TCM) cells (CD45RA- CCR7+), effector memory T (TEM) cells (CD45RA- CCR7-), and effector memory T with CD45RA expression (TEMRA) cells (CD45RA+ CCR7-) was calculated out of CD8+ T cells from PBMC, SN, and tumor. The data are means with the error bars indicating SEM. Kruskal-Wallis was used as the statistical test. * p<0.05, **p<0.01, ***p<0.001, ****p<0.0001. (TIF) [file pone.0200079.s001.tif]

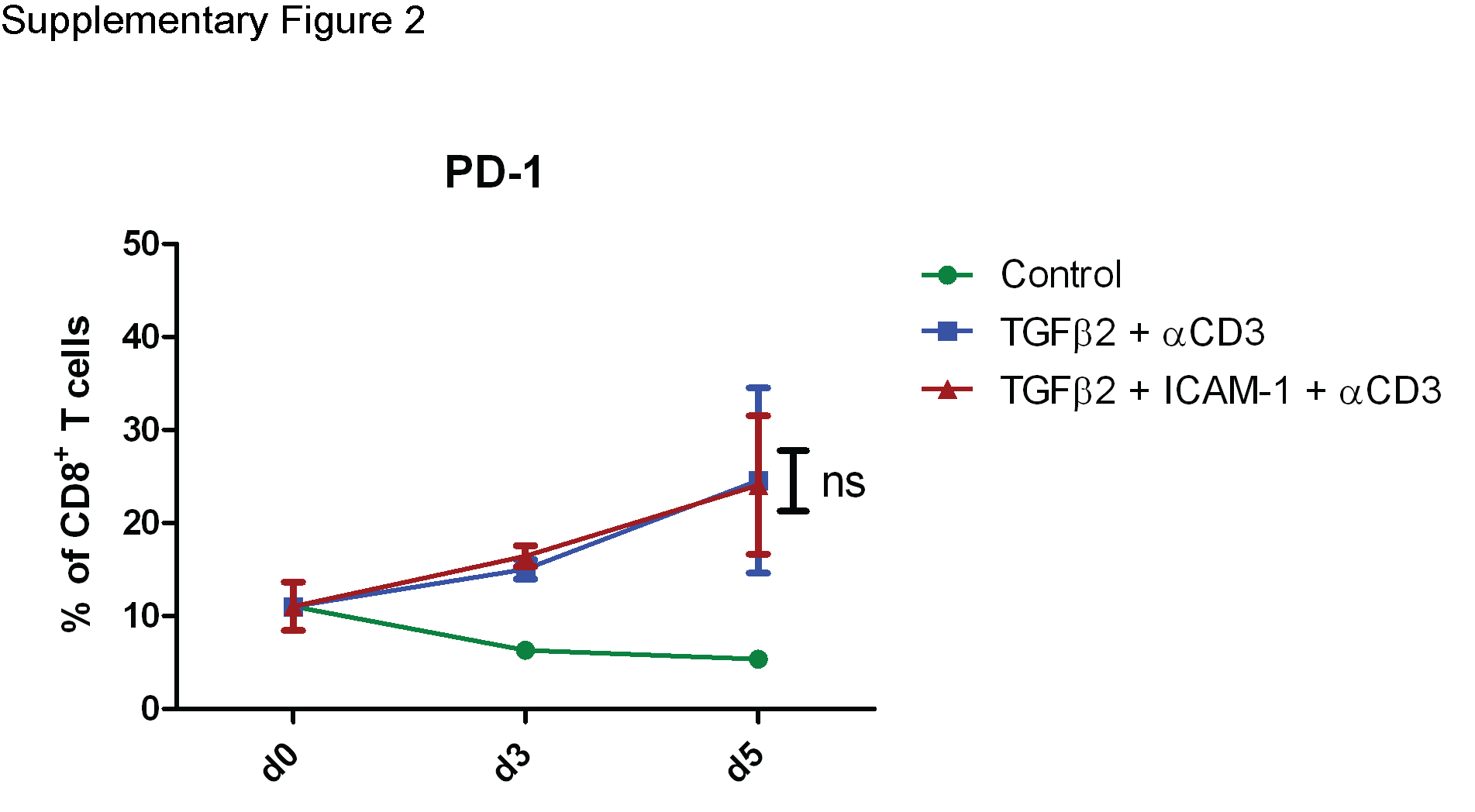

Supplement: S2 Fig — CD8+ T cells isolated from healthy donors were cultured in vitro in the presence of TGFβ2, anti-CD3 stimulating antibody, with or without ICAM-1 Fc chimera. Flow cytometry analysis of PD-1 expression on CD8+ T cells was performed at baseline (day 0), day 3, and day 5. The frequency of PD-1-expressing cells was counted out of CD8+ T cells. The data are means with error bars indicating SEM. One-way repeated-measure ANOVA was used as the statistical test. * p<0.05, **p<0.01, ***p<0.001, ****p<0.0001. (TIF) [file pone.0200079.s002.tif]
